# Supplementary material for: Molecular Pathogenesis of Pancreatic Ductal Adenocarcinoma: Impact of miR-30c-5p and miR-30c-2-3p Regulation on Oncogenic Genes
Source: Cancers (Basel). 2020 Sep 23;12(10):2731. doi: 10.3390/cancers12102731 (PMC7598296; doi:10.3390/cancers12102731)
Supplement: Supplementary file 1 [file cancers-12-02731-s001.pdf]

cancers

MDPI

Molecular Pathogenesis of Pancreatic Ductal Adenocarcinoma: Impact of *miR-30c-5p* and *miR-30c-2-3p* Regulation on Oncogenic Genes

Takako Tanaka, Reona Okada, Yuto Hozaka, Masumi Wada, Shogo Moriya, Souichi Satake, Tetsuya Idichi, Hiroshi Kurahara, Takao Ohtsuka and Naohiko Seki

Table S1. Clinical samples' patient characteristics

No.	Age	Sex	Location	T	N	M	stage	Differentiation
Tumor 1	42	M	pancreas head	3	1	0	2B	well-moderate
Tumor 2	44	M	pancreas head	3	1	0	2B	modetrate
Tumor 3	76	M	pancreas head	3	1	0	2B	mode-poor
Tumor 4	67	M	pancreas head	3	1	0	2B	moderate
Tumor 5	78	F	pancreas head	3	0	0	2A	Papillary
Tumor 6	66	M	pancreas head	3	1	0	2B	moderate
Tumor 7	58	F	pancreas head	3	0	0	2A	moderate
Tumor 8	42	F	pancreas head	3	1	0	2B	well
Tumor 9	65	M	pancreas body	3	1	1	4	well
Tumor 10	56	F	pancreas body	3	1	0	2B	well
Tumor 11	70	M	pancreas head	3	1	0	2B	well
Tumor 12	63	F	pancreas head	3	0	0	2A	poor
Tumor 13	52	M	pancreas body	3	1	0	2B	well
Tumor 14	56	M	pancreas body	3	1	1	4	well
Tumor 15	65	M	pancreas body	2	0	0	1B	well
Tumor 16	78	F	pancreas head	3	1	0	2B	well+poor
Tumor 17	50	F	pancreas head	3	0	1	4	neuro
Tumor 18	66	F	pancreas head	3	0	0	2A	well
Tumor 19	66	F	pancreas head	3	1	0	2B	well+poor
Tumor 20	67	M	pancreas tail	0	0	0	0	no date
Tumor 21	74	M	pancreas tail	3	0	0	2A	well
Tumor 22	74	F	pancreas body	3	1	0	2B	well
Tumor 23	65	F	pancreas head	3	1	0	2B	well+poor
Tumor 24	78	M	pancreas head	3	0	0	2A	well
Tumor 25	71	F	pancreas body	3	0	0	2A	well+poor
Tumor 26	64	M	pancreas head	3	0	0	2A	moderate
Tumor 27	72	F	pancreas tail	3	1	0	2B	moderate

Features of patients in noncancerous pancreatic tissues.

No.	Age	Sex
Normal 1	65	F
Normal 2	58	F
Normal 3	77	F
Normal 4	67	M
Normal 5	42	F
Normal 6	65	F
Normal 7	60	M
Normal 8	56	F
Normal 9	67	M
Normal 10	85	F
Normal 11	66	F
Normal 12	65	F
Normal 13	76	M
Normal 14	71	F
Normal 15	64	M

Normal 16	72	F

Table S2. Putative target genes by *miR-30c-5p* and *miR− 30c−2−3p* regulation in PDAC cells.

Putative target genes by *miR-30c-5p* regulation in PDAC cells.							
**Entrez Gene ID**	**Gene Symbol**	**Gene name**	**GEO FC**	**GEO log2 FC**	***PANC-1 miR-30c-5p* transfectant FC**	***PANC-1 miR-30c-5p* transfectant log2 FC**	**Total binding sites**
7534	*YWHAZ*	tyrosine 3-monooxygenase/tryptophan 5-monooxygenase activation protein, zeta polypeptide	2.107888	1.075798213	−1.8025312	−0.850024	1
776	*CACNA1D*	calcium channel, voltage−dependent, L type, alpha 1D subunit	2.1054492	1.074128067	−1.8065524	−0.853239	1
865	*CBFB*	core−binding factor, beta subunit	2.2276592	1.155528538	−2.3320081	−1.221573	2
79026	*AHNAK*	AHNAK nucleoprotein	2.1226394	1.085859303	−2.5719824	−1.362881	1
5357	*PLS1*	plastin 1	2.1775904	1.122732612	−1.5772574	−0.657418	1
55450	*CAMK2N1*	calcium/calmodulin−dependent protein kinase II inhibitor 1	2.8895435	1.530841589	−1.7339722	−0.794081	1
285761	*DCBLD1*	discoidin, CUB and LCCL domain containing 1	3.3312216	1.736051328	−1.7587084	−0.813653	2
4547	*MTTP*	microsomal triglyceride transfer protein	2.0088177	1.006346646	−2.8433008	−1.507567	1
114907	*FBXO32*	F−box protein 32	3.750639	1.90713641	−1.6121457	−0.688982	3
23333	*DPY19L1*	dpy−19−like 1 (C. elegans)	2.8526917	1.512323838	−2.2663465	−1.180368	3
3977	*LIFR*	leukemia inhibitory factor receptor alpha	2.258954	1.175654893	−2.001093	−1.000788	2
4907	*NT5E*	5'−nucleotidase, ecto (CD73)	2.9813142	1.575948429	−3.7137697	−1.892884	2
25963	*TMEM87A*	transmembrane protein 87A	2.0282607	1.020243099	−1.6889185	−0.7561	1
84230	*LRRC8C*	leucine rich repeat containing 8 family, member C	2.0576832	1.041020883	−1.5065249	−0.591225	2
54855	*FAM46C*	family with sequence similarity 46, member C	2.5071456	1.326045782	−1.8364283	−0.876903	2
53616	*ADAM22*	ADAM metallopeptidase domain 22	2.3424919	1.228044059	−1.8035026	−0.850801	1
90	*ACVR1*	activin A receptor, type I	2.838798	1.505280195	−1.5178666	−0.602045	1
54492	*NEURL1B*	neuralized homolog 1B (Drosophila)	2.3553534	1.23594354	−1.517008	−0.601229	1
25960	*GPR124*	G protein−coupled receptor 124	2.2712364	1.183477876	−2.3105528	−1.208238	1
5783	*PTPN13*	protein tyrosine phosphatase, non−receptor type 13 (APO−1/CD95 (Fas)−associated phosphatase)	2.0204637	1.014686432	−1.8147175	−0.859745	2
2494	*NR5A2*	nuclear receptor subfamily 5, group A, member 2	2.9269483	1.549397263	−1.6877522	−0.755103	2
57157	*PHTF2*	putative homeodomain transcription factor 2	2.4678419	1.303249973	−1.7268459	−0.788139	3
10484	*SEC23A*	Sec23 homolog A (S. cerevisiae)	2.3764641	1.248816608	−4.183498	−2.06471	2
3680	*ITGA9*	integrin, alpha 9	2.3379743	1.225259071	−1.6452351	−0.718294	1
10672	*GNA13*	guanine nucleotide binding protein (G protein), alpha 13	2.157417	1.109305057	−1.8154717	−0.859829	4
63895	*PIEZO2*	piezo−type mechanosensitive ion channel component 2	2.6537354	1.408024529	−2.4506588	−1.29317	1
5738	*PTGFRN*	prostaglandin F2 receptor inhibitor	2.642839	1.40208854	−5.7623205	−2.52665	3
1756	*DMD*	dystrophin	2.4918914	1.317241195	−1.5377088	−0.620782	1

9859	*CEP170*	centrosomal protein 170kDa	3.615709	1.854278571	−1.8422364	−0.881458	1
9315	*NREP*	neuronal regeneration related protein	4.4351254	2.148974896	−2.4860835	−1.313875	1
115908	*CTHRC1*	collagen triple helix repeat containing 1	21.539711	4.428926988	−3.2357185	−1.694086	1
285598	*ARL10*	ADP−ribosylation factor−like 10	2.0289125	1.020706648	−1.6264279	−0.701707	5
860	*RUNX2*	runt−related transcription factor 2	7.6337624	2.932394284	−2.316619	−1.212021	4
6443	*SGCB*	sarcoglycan, beta (43kDa dystrophin−associated glycoprotein)	2.172902	1.119623109	−3.9752877	−1.991059	1
1734	*DIO2*	deiodinase, iodothyronine, type II	4.1299953	2.04614014	−2.2864702	−1.193122	1
222658	*KCTD20*	potassium channel tetramerization domain containing 20	2.6426406	1.401980231	−1.8270454	−0.869513	1
5396	*PRRX1*	paired related homeobox 1	6.295065	2.654221275	−2.6690764	−1.416341	1
154141	*MBOAT1*	membrane bound O−acyltransferase domain containing 1	2.0484128	1.034506479	−1.6067419	−0.684138	1
29887	*SNX10*	sorting nexin 10	2.3028336	1.203410167	−1.7153641	−0.778515	1
8417	*STX7*	syntaxin 7	2.1770043	1.122344257	−2.014982	−1.010767	4
8819	*SAP30*	Sin3A−associated protein, 30kDa	2.1350708	1.094283911	−3.6536663	−1.869345	2
5159	*PDGFRB*	platelet−derived growth factor receptor, beta polypeptide	3.4799414	1.799063012	−3.8238856	−1.8839725	1
1131	*CHRM3*	cholinergic receptor, muscarinic 3	2.8919995	1.532067303	−1.8628036	−0.897476	2
7431	*VIM*	vimentin	2.1574442	1.109323246	−3.127566	−1.64504	2
170954	*PPP1R18*	protein phosphatase 1, regulatory subunit 18	2.5516028	1.351403767	−1.7732465	−0.826393	3
54749	*EPDR1*	ependymin related 1	2.011554	1.008310468	−2.9045637	−1.538322	3
54566	*EPB41L4B*	erythrocyte membrane protein band 4.1 like 4B	3.2564375	1.703294538	−2.165876	−1.0574225	1
60481	*ELOVL5*	ELOVL fatty acid elongase 5	3.1485033	1.654666179	−1.7085098	−0.772739	1
4325	*MMP16*	matrix metallopeptidase 16 (membrane−inserted)	2.0137687	1.009897986	−2.4893477	−1.315768	1
8829	*NRP1*	neuropilin 1	2.4660127	1.30218023	−1.9796517	−0.985247	1
57482	*KIAA1211*	KIAA1211	3.3908448	1.761644753	−1.854175	−0.890777	1

Putative target genes by *miR− 30c−2−3p* regulation in PDAC cells.

Entrez Gene ID	Gene Symbol	Gene Name	GEO FC	GEO log2 FC	*PANC−1 miR−30c−2−3p* transfectant FC	*PANC−1 miR−30c−2−3p* transfectant log2 FC	Total binding sites
23052	*ENDOD1*	endonuclease domain containing 1	2.2613354	1.17717499	−1.5562582	−0.638082	1
2152	*F3*	coagulation factor III (thromboplastin, tissue factor)	2.6254416	1.3925601	−1.6159481	−0.692381	1
29766	*TMOD3*	tropomodulin 3 (ubiquitous)	2.0454962	1.03245086	−2.7957864	−1.483254	2
3675	*ITGA3*	integrin, alpha 3 (antigen CD49C, alpha 3 subunit of VLA−3 receptor)	2.5118082	1.32872631	−2.564058	−1.358429	2
7153	*TOP2A*	topoisomerase (DNA) II alpha 170kDa	2.8882535	1.53019737	−2.2842295	−1.191708	1
9208	*LRRFIP1*	leucine rich repeat (in FLII) interacting protein 1	2.648011	1.40490912	−2.430869	−1.281472	2
6237	*RRAS*	related RAS viral (r−ras) oncogene homolog	2.2145765	1.14703083	−1.8885567	−0.917284	1
9603	*NFE2L3*	nuclear factor, erythroid 2−like 3	2.8481824	1.51004154	−2.1272888	−1.089016	3

11098	*PRSS23*	protease, serine, 23	2.7526407	1.46081631	−1.9241873	−0.944249	1
3556	*IL1RAP*	interleukin 1 receptor accessory protein	2.0077293	1.00556477	−1.7964197	−0.825645	1
129642	*MBOAT2*	membrane bound O−acyltransferase domain containing 2	2.9205017	1.54621622	−3.6262991	−1.858498	2
27042	*DIEXF*	digestive organ expansion factor homolog (zebrafish)	2.1144078	1.08025365	−1.6244218	−0.699926	5
130340	*AP1S3*	adaptor−related protein complex 1, sigma 3 subunit	2.0921135	1.06496112	−4.180721	−2.04955067	3
6307	*MSMO1*	methylsterol monooxygenase 1	2.137287	1.09578065	−3.8763406	−1.954695	3
8553	*BHLHE40*	basic helix−loop−helix family, member e40	3.2192662	1.68673188	−1.5556555	−0.637523	1
23603	*CORO1C*	coronin, actin binding protein, 1C	2.9602175	1.56570318	−2.8987157	−1.535414	1
6241	*RRM2*	ribonucleotide reductase M2	2.2445	1.1663941	−2.0015752	−1.001136	1
64108	*RTP4*	receptor (chemosensory) transporter protein 4	2.265131	1.17959449	−5.888388	−2.557873	1
29108	*PYCARD*	PYD and CARD domain containing	2.0466015	1.03323022	−2.5309663	−1.339688	1
131566	*DCBLD2*	discoidin, CUB and LCCL domain containing 2	3.1744227	1.66649425	−2.2274199	−1.155374	2
84441	*MAML2*	mastermind−like 2 (Drosophila)	2.519923	1.33337965	−1.8320524	−0.873461	2
7414	*VCL*	vinculin	2.0701628	1.04974423	−1.788988	−0.839144	3
22925	*PLA2R1*	phospholipase A2 receptor 1, 180kDa	2.3120809	1.20919188	−1.9828366	−0.987566	1
22822	*PHLDA1*	pleckstrin homology−like domain, family A, member 1	3.0949705	1.62992566	−2.4945047	−1.318753	1
55107	*ANO1*	anoctamin 1, calcium activated chloride channel	7.8534274	2.97332241	−3.279582	−1.713512	1
50515	*CHST11*	carbohydrate (chondroitin 4) sulfotransferase 11	2.9458146	1.55866663	−1.8003976	−0.848316	1
684	*BST2*	bone marrow stromal cell antigen 2	2.957019	1.56414351	−2.9614215	−1.564264	1
1942	*EFNA1*	ephrin−A1	2.0360727	1.02578908	−1.870977	−0.903792	2
4015	*LOX*	lysyl oxidase	8.548389	3.09565256	−1.5591584	−0.640768	2
10379	*IRF9*	interferon regulatory factor 9	2.1249146	1.08740486	−1.5612102	−0.642665	1
355	*FAS*	Fas cell surface death receptor	2.9723217	1.57159027	−2.8188543	−1.495109	1
29	*ABR*	active BCR−related	2.08959	1.0632199	−2.49846555	−1.3182065	1
284723	*SLC25A34*	solute carrier family 25, member 34	2.1012342	1.07123697	−2.2505753	−1.170294	2
57198	*ATP8B2*	ATPase, aminophospholipid transporter, class I, type 8B, member 2	2.2136514	1.14642805	−3.3745415	−1.754692	1
25878	*MXRA5*	matrix−remodelling associated 5	5.2305136	2.38695262	−4.461383	−2.157491	1
3656	*IRAK2*	interleukin−1 receptor−associated kinase 2	2.2026823	1.13926143	−3.5588243	−1.831401	1
203068	*TUBB*	tubulin, beta class I	2.081564	1.05766792	−1.74200455	−0.7978345	1
91404	*SESTD1*	SEC14 and spectrin domains 1	2.61934	1.38920334	−1.713972	−0.777344	1
2053	*EPHX2*	epoxide hydrolase 2, cytoplasmic	2.686746	1.42585994	−1.5061291	−0.590846	1
2069	*EREG*	epiregulin	2.4593692	1.29828833	−3.0630414	−1.614965	1
56937	*PMEPA1*	prostate transmembrane protein, androgen induced 1	4.4851875	2.1651683	−1.6591595	−0.7277565	1
9770	*RASSF2*	Ras association (RalGDS/AF−6) domain family member 2	2.853415	1.51268959	−2.571092	−1.362381	2
5339	*PLEC*	plectin	2.1774795	1.12265914	−2.614268	−1.386407	3
558	*AXL*	AXL receptor tyrosine kinase	2.9549663	1.56314168	−2.58509	−1.370215	3
57713	*SFMBT2*	Scm−like with four mbt domains 2	2.4176946	1.27363202	−2.0261168	−1.018717	4

2000	*ELF4*	E74−like factor 4 (ets domain transcription factor)	2.6211767	1.39021461	−1.7536374	−0.810351	2
11213	*IRAK3*	interleukin−1 receptor−associated kinase 3	3.9714346	1.98966025	−2.6676056	−1.415546	1
162073	*ITPRIPL2*	inositol 1,4,5−trisphosphate receptor interacting protein−like 2	2.0831883	1.05879325	−1.7624962	−0.81762	4
57125	*PLXDC1*	plexin domain containing 1	3.7879417	1.92141413	−1.5919814	−0.6693795	1
10135	*NAMPT*	nicotinamide phosphoribosyltransferase	2.0969985	1.06832583	−1.5757401	−0.65603	1
5912	*RAP2B*	RAP2B, member of RAS oncogene family	2.7403688	1.45437006	−4.9478607	−2.306805	2
3777	*KCNK3*	potassium channel, subfamily K, member 3	2.4246292	1.27776413	−2.9468246	−1.559161	4
9123	*SLC16A3*	solute carrier family 16 (monocarboxylate transporter), member 3	6.238611	2.64122485	−1.5706434	−0.651356	2
57619	*SHROOM3*	shroom family member 3	2.1864073	1.12856218	−1.93803985	−0.948166	4
23043	*TNIK*	TRAF2 and NCK interacting kinase	2.1772404	1.12250071	−2.91195425	−1.485177	2
1296	*COL8A2*	collagen, type VIII, alpha 2	3.9824197	1.99364527	−3.2619098	−1.6652685	2
59339	*PLEKHA2*	pleckstrin homology domain containing, family A (phosphoinositide binding specific) member 2	2.0061517	1.0044307	−2.3900883	−1.257064	1
710	*SERPING1*	serpin peptidase inhibitor, clade G (C1 inhibitor), member 1	2.8698225	1.52096151	−2.8072548	−1.48916	4
4939	*OAS2*	2'−5'−oligoadenylate synthetase 2, 69/71kDa	2.6352875	1.39796036	−2.34519413	−1.16816167	2
85415	*RHPN2*	rhophilin, Rho GTPase binding protein 2	2.1334422	1.09318303	−1.80605875	−0.8398745	2
368	*ABCC6*	ATP−binding cassette, sub−family C (CFTR/MRP), member 6	2.0255477	1.01831206	−5.51934563	−2.20208975	1
55083	*KIF26B*	kinesin family member 26B	7.422415	2.89188867	−1.8272907	−0.869706	2
340024	*SLC6A19*	solute carrier family 6 (neutral amino acid transporter), member 19	2.0228834	1.01641316	−1.5889257	−0.668052	1
3718	*JAK3*	Janus kinase 3	2.0072212	1.00519961	−12.895146	−3.688756	3
7220	*TRPC1*	transient receptor potential cation channel, subfamily C, member 1	2.8364346	1.5040786	−3.6420674	−1.864758	1
4175	*MCM6*	minichromosome maintenance complex component 6	2.0213459	1.01531622	−2.1289797	−1.090162	1
219285	*SAMD9L*	sterile alpha motif domain containing 9−like	2.292748	1.19707779	−1.6227703	−0.698459	1
1999	*ELF3*	E74−like factor 3 (ets domain transcription factor, epithelial−specific)	2.274228	1.1853769	−2.3688393	−1.24418	1
10846	*PDE10A*	phosphodiesterase 10A	2.24667	1.16778823	−1.66160585	−0.729165	1
3092	*HIP1*	huntingtin interacting protein 1	3.1944785	1.67558043	−8.986034	−3.167685	3
1122	*CHML*	choroideremia−like (Rab escort protein 2)	2.3433568	1.22857664	−6.369226	−2.671118	2
80005	*DOCK5*	dedicator of cytokinesis 5	2.30235	1.20310717	−1.5297543	−0.6133	2
9388	*LIPG*	lipase, endothelial	2.760285	1.46481723	−4.932008	−2.302175	3
6581	*SLC22A3*	solute carrier family 22 (organic cation transporter), member 3	2.1399078	1.09754864	−1.6263621	−0.701649	3
26112	*CCDC69*	coiled−coil domain containing 69	2.4701278	1.30458569	−1.514434	−0.598779	1
9902	*MRC2*	mannose receptor, C type 2	2.612301	1.38532114	−2.5926282	−1.374415	1
9456	*HOMER1*	homer homolog 1 (Drosophila)	2.461455	1.29951136	−1.6948876	−0.76119	1
51278	*IER5*	immediate early response 5	2.0735698	1.05211661	−2.4117854	−1.270102	5

10100	*TSPAN2*	tetraspanin 2	2.6602573	1.41156579	−2.5570564	−1.354484	3
3976	*LIF*	leukemia inhibitory factor	2.2362914	1.16110819	−2.25570925	−1.1157615	2
2687	*GGT5*	gamma−glutamyltransferase 5	2.1869118	1.12889504	−3.0339217	−1.601184	1
4148	*MATN3*	matrilin 3	4.523766	2.17752431	−2.1714654	−1.118669	1
5069	*PAPPA*	pregnancy−associated plasma protein A, pappalysin 1	3.3934433	1.76274991	−1.6303263	−0.705161	2
344558	*SH3RF3*	SH3 domain containing ring finger 3	2.101751	1.07159176	−2.0393922	−1.028139	2
5476	*CTSA*	cathepsin A	2.1106308	1.07767424	−3.55539295	−1.820275	1
84168	*ANTXR1*	anthrax toxin receptor 1	7.4770546	2.90247007	−2.7002661	−1.433102	1
9830	*TRIM14*	tripartite motif containing 14	2.3578706	1.23748455	−2.26918445	−1.124839	2
875	*CBS*	cystathionine−beta−synthase	4.0390577	2.01401876	−2.9737785	−1.572297	7
1794	*DOCK2*	dedicator of cytokinesis 2	2.2617252	1.17742365	−1.5354911	−0.6187	1
5272	*SERPINB9*	serpin peptidase inhibitor, clade B (ovalbumin), member 9	3.1824975	1.67015938	−2.953741	−1.562543	3
4065	*LY75*	lymphocyte antigen 75	3.5984533	1.84737694	−3.0177822	−1.593489	1
871	*SERPINH1*	serpin peptidase inhibitor, clade H (heat shock protein 47), member 1, (collagen binding protein 1)	4.0745363	2.02663588	−2.719987	−1.4436	2
4638	*MYLK*	myosin light chain kinase	2.6462674	1.40395885	−2.41026485	−1.2675595	2
8324	*FZD7*	frizzled family receptor 7	3.0004196	1.58516427	−1.7274305	−0.788628	2
9644	*SH3PXD2A*	SH3 and PX domains 2A	3.098084	1.63137626	−2.3469895	−1.230165	1
10052	*GJC1*	gap junction protein, gamma 1, 45kDa	2.4474685	1.29129029	−1.7094983	−0.773573	1
10687	*PNMA2*	paraneoplastic Ma antigen 2	4.17114	2.06044174	−6.3219113	−2.660361	3
7162	*TPBG*	trophoblast glycoprotein	3.1523435	1.65642475	−1.7106761	−0.774567	2
56648	*EIF5A2*	eukaryotic translation initiation factor 5A2	2.7315953	1.44974376	−3.0672145	−1.616929	4
83742	*MARVELD1*	MARVEL domain containing 1	2.2957945	1.19899351	−2.5303726	−1.33935	1
130271	*PLEKHH2*	pleckstrin homology domain containing, family H (with MyTH4 domain) member 2	2.3987784	1.26229989	−1.818772	−0.862965	2
659	*BMPR2*	bone morphogenetic protein receptor, type II (serine/threonine kinase)	2.4564056	1.2965488	−1.6440841	−0.717284	1
2274	*FHL2*	four and a half LIM domains 2	3.8398573	1.9410527	−1.65125545	−0.7195805	2
56243	*KIAA1217*	KIAA1217	2.6544228	1.40839818	−2.2281299	−1.155833	1
2151	*F2RL2*	coagulation factor II (thrombin) receptor−like 2	5.936649	2.56964882	−7.8835106	−2.978838	4
4053	*LTBP2*	latent transforming growth factor beta binding protein 2	3.249144	1.70005968	−2.5622756	−1.357426	2
1690	*COCH*	cochlin	3.8746495	1.95406581	−3.2093477	−1.68228	1
4093	*SMAD9*	SMAD family member 9	2.2520816	1.1712591	−2.51712345	−1.3037185	2
55243	*KIRREL*	kin of IRRE like (Drosophila)	3.0286162	1.59865876	−2.18751375	−1.12549	2
284611	*FAM102B*	family with sequence similarity 102, member B	2.593979	1.3751668	−5.2071325	−2.375539	2
57722	*IGDCC4*	immunoglobulin superfamily, DCC subclass, member 4	2.6780534	1.42118473	−2.9324749	−1.552119	3
718	*C3*	complement component 3	4.95801	2.30976118	−1.8616517	−0.896583	6
22797	*TFEC*	transcription factor EC	3.1502035	1.65544503	−2.4804018	−1.310574	1

6533	*SLC6A6*	solute carrier family 6 (neurotransmitter transporter), member 6	6.4249697	2.68368965	−4.171237	−2.060475	3
5740	*PTGIS*	prostaglandin I2 (prostacyclin) synthase	4.4972715	2.16904998	−5.5244417	−2.465829	4
7070	*THY1*	Thy−1 cell surface antigen	5.038337	2.33294762	−1.9090989	−0.932892	1
867	*CBL*	Cbl proto−oncogene, E3 ubiquitin protein ligase	2.0819829	1.05795822	−1.7303138	−0.791034	2
84662	*GLIS2*	GLIS family zinc finger 2	3.0599735	1.61351916	−2.0631127	−1.044823	3
51303	*FKBP11*	FK506 binding protein 11, 19 kDa	2.5678456	1.36055846	−2.53125675	−1.3380525	1
6480	*ST6GAL1*	ST6 beta−galactosamide alpha−2,6−sialyltranferase 1	2.023059	1.01653839	−5.692959	−2.509179	1
2266	*FGG*	fibrinogen gamma chain	3.3034732	1.72398364	−3.9274867	−1.973606	2
285203	*EOGT*	EGF domain−specific O−linked N−acetylglucosamine (GlcNAc) transferase	2.3285272	1.21941773	−1.7727354	−0.825977	2
1728	*NQO1*	NAD(P)H dehydrogenase, quinone 1	4.5524726	2.18665033	−1.7585198	−0.814362	1
3613	*IMPA2*	inositol(myo)−1(or 4)−monophosphatase 2	2.8482149	1.510058	−3.329885	−1.7180095	3
4330	*MN1*	meningioma (disrupted in balanced translocation) 1	3.5127406	1.81259704	−1.570467	−0.651194	1
1959	*EGR2*	early growth response 2	3.8833003	1.95728328	−2.5051596	−1.324903	1
686	*BTD*	biotinidase	2.1993654	1.13708731	−2.089345	−1.063051	11
11037	*STON1*	stonin 1	3.027899	1.59831708	−2.28250615	−1.1797175	1
60681	*FKBP10*	FK506 binding protein 10, 65 kDa	2.018864	1.01354373	−3.4984043	−1.806697	1
79627	*OGFRL1*	opioid growth factor receptor−like 1	2.0309052	1.0221229	−2.8983648	−1.535239	1
493869	*GPX8*	glutathione peroxidase 8 (putative)	4.398069	2.13687024	−1.5208063	−0.604836	3
89796	*NAV1*	neuron navigator 1	2.4964359	1.31986986	−4.78107215	−2.2445055	4
144165	*PRICKLE1*	prickle homolog 1 (Drosophila)	3.1929393	1.67488513	−3.0424352	−1.605227	1
4811	*NID1*	nidogen 1	3.1419613	1.65166541	−7.9136095	−2.984336	2
57616	*TSHZ3*	teashirt zinc finger homeobox 3	2.1751404	1.12110853	−4.710902	−2.236003	1
57333	*RCN3*	reticulocalbin 3, EF−hand calcium binding domain	2.312374	1.20937476	−1.633107	−0.707619	1
5010	*CLDN11*	claudin 11	5.816361	2.54011681	−2.4935548	−1.318204	2
3667	*IRS1*	insulin receptor substrate 1	2.6697042	1.4166799	−1.9530267	−0.965712	2
84706	*GPT2*	glutamic pyruvate transaminase (alanine aminotransferase) 2	2.363553	1.24095722	−4.603926	−2.202865	3
3212	*HOXB2*	homeobox B2	3.2313602	1.69214158	−2.760372	−1.464863	1
10855	*HPSE*	heparanase	2.1441035	1.10037455	−1.6127669	−0.689538	1
3111	*HLA−DOA*	major histocompatibility complex, class II, DO alpha	2.0180554	1.01296578	−2.9175372	−1.544751	1
3487	*IGFBP4*	insulin−like growth factor binding protein 4	3.1715395	1.66518331	−1.9788606	−0.98467	3
23301	*EHBP1*	EH domain binding protein 1	2.1654549	1.11467013	−1.6429753	−0.710911	1
285590	*SH3PXD2B*	SH3 and PX domains 2B	2.481224	1.31105198	−5.5057864	−2.460949	1
388610	*TRNP1*	TMF1−regulated nuclear protein 1	2.1865497	1.12865614	−1.9689997	−0.977463	2
8477	*GPR65*	G protein−coupled receptor 65	2.574515	1.36430068	−1.9066031	−0.931005	2
65108	*MARCKSL1*	MARCKS−like 1	2.511199	1.32837636	−3.3468802	−1.742817	3
91663	*MYADM*	myeloid−associated differentiation marker	2.2757297	1.18632921	−1.5950052	−0.673561	2

4499	*MT1M*	metallothionein 1M	2.7478464	1.45830136	−2.3982008	−1.261952	1
83879	*CDCA7*	cell division cycle associated 7	2.4945168	1.31876039	−3.52452985	−1.733681	1
5481	*PPID*	peptidylprolyl isomerase D	2.1669471	1.11566393	−1.7721004	−0.82546	1
8459	*TPST2*	tyrosylprotein sulfotransferase 2	3.4068055	1.76841958	−1.973812	−0.980985	1
2043	*EPHA4*	EPH receptor A4	2.785011	1.47768303	−3.0478623	−1.607798	1
84056	*KATNAL1*	katanin p60 subunit A−like 1	2.2414992	1.16446398	−1.9797919	−0.985349	3
54796	*BNC2*	basonuclin 2	4.100778	2.03589764	−5.485393	−2.455595	2
9075	*CLDN2*	claudin 2	2.3763666	1.24875742	−1.5970572	−0.675416	1
10962	*MLLT11*	myeloid/lymphoid or mixed−lineage leukemia (trithorax homolog, Drosophila); translocated to, 11	2.389446	1.25667616	−4.943322	−2.305481	1
5552	*SRGN*	serglycin	2.812706	1.49195876	−2.3373752	−1.224889	1
972	*CD74*	CD74 molecule, major histocompatibility complex, class II invariant chain	2.9994717	1.58470842	−1.5113016	−0.595792	1
50807	*ASAP1*	ArfGAP with SH3 domain, ankyrin repeat and PH domain 1	2.6920624	1.42871185	−1.5026035	−0.587464	1
2869	*GRK5*	G protein−coupled receptor kinase 5	2.0465665	1.03320555	−2.7596712	−1.464496	2
10365	*KLF2*	Kruppel−like factor 2 (lung)	2.0431898	1.03082323	−2.627674	−1.393786	3
11317	*RBPJL*	recombination signal binding protein for immunoglobulin kappa J region−like	5.1099987	2.35332292	−1.981117	−0.986314	1
495	*ATP4A*	ATPase, H+/K+ exchanging, alpha polypeptide	2.9357285	1.55371855	−1.87758	−0.908874	1

Table S3. The expression levels of protein

Gene	Antibody	Antibody staining			
		High	Medium	Low	Not detected
YWHAZ	CAB005065	12/12	–	–	–
CBFB	HPA038852	–	3/12	6/12	3/12
F3	CAB009438	4/12	6/12	2/12	
TMOD3	HPA001849	9/10	1/10	–	–
NFE2L3	HPA055889	–	–	–	11/11
ENDOD1	HPA008932	2/11	5/11	3/11	1/11
ITGA3	HPA008572	7/11	3/11	–	1/11
RRAS	CAB10160	–	2/9	4/9	3/9
PRSS23	HPA030591	–	–	1/11	10/11
TOP2A	HPA006458	5/11	6/11	–	–

BHLHE40	HPA028922	2/11	4/11	1/11	4/11
CHST11	HPA052828	–	2/8	1/8	5/8
IL1RAP	HPA035293	–	4/12	2/12	6/12
DIEXF	HPA026640	4/11	5/11	1/11	1/11
CORO1C	HPA041737	3/12	9/12	–	–
RTP4	HPA064887	–	8/9	–	1/9
AP1S3	HPA066782	–	–	–	8/8
LRRFIP1	HPA006979	5/10	5/10	–	–

Table S4. Gene Ontology classification.

Term	Annotation	hyp_pval	Genes
protein binding,cytoplasm,nucleus,DNA binding,negative regulation of transcription by RNA polymerase II, regulation of transcription by RNA polymerase II,regulation of transcription, DNA-templated, RNA polymerase II cis−regulatory region sequence-specific DNA binding, DNA−binding transcription repressor activity, RNA polymerase II-specific	GO:0005515,GO:0005737,GO:0005634, GO:0003677,GO:0000122,GO:0006357, GO:0006355,GO:0000978,GO:0001227	1.35E−06	*NFE2L3,LRRFIP1,BHLHE40*
protein binding,cytoplasm,protein homodimerization activity,nucleus,DNA binding	GO:0005515,GO:0005737,GO:0042803, GO:0005634,GO:0003677	6.20E−06	*LRRFIP1,BHLHE40,TOP2A*
protein binding,nucleus,DNA binding,negative regulation of transcription by RNA polymerase II, regulation of transcription by RNA polymerase II	GO:0005515,GO:0005634,GO:0003677, GO:0000122,GO:0006357	6.19E−05	*NFE2L3,LRRFIP1,CBFB,BHLHE40*
extracellular exosome,protein binding,focal adhesion	GO:0070062,GO:0005515,GO:0005925	0.0002313 24	*YWHAZ,RRAS,ITGA3*
positive regulation of NF-kappaB transcription factor activity	GO:0051092	0.0001972 63	*LRRFIP1,IL1RAP,RIP4*
protein binding,cytoplasm,nucleus,DNA binding	GO:0005515,GO:0005737,GO:0005634,GO:0 003677	0.0003517 21	*NFE2L3,LRRFIP1,BHLHE40,TOP2A*
protein binding,protein domain specific binding	GO:0005515,GO:0019904	0.0007610 23	*BHLHE40,YWHAZ,ITGA3*
Cytokine-mediated signaling pathway	GO:0019221	0.0010900 9	*YWHAZ,IL1RAP,F3*

protein binding,protein heterodimerization activity	GO:0005515,GO:0046982	0.0010264 9	*BHLHE40,TOP2A,ITGA3*
protein binding,nucleus,DNA binding	GO:0005515,GO:0005634,GO:0003677	0.0045147 2	*NFE2L3,LRRFIP1,CBFB,BHLHE40,TOP2A*
membrane,hydrolase activity	GO:0016020,GO:0016787	0.0100205	*ENDOD1,RRAS,IL1RAP*
extracellular region,membrane	GO:0005576,GO:0016020	0.0152731	*ENDOD1,IL1RAP,F3*

Table S5. Reagents used in this study.

TaqMan Primers And Probes	Assay ID		Company
hsa-miR-30c-5p	000419		Applied Biosystems, Waltham, MA, USA
hsa-miR-30c-2-3p	002110		Applied Biosystems, Waltham, MA, USA
RNU48	001006		Applied Biosystems, Waltham, MA, USA
TOP2A	Hs01032137		Applied Biosystems, Waltham, MA, USA
GUSB	Hs99999908_m1		Applied Biosystems, Waltham, MA, USA
**Pre-miR miRNA Presursors**	**Assay ID**	**Concentration**	
hsa-miR-30c-5p	PM11060	10 nM	Applied Biosystems, Waltham, MA, USA
hsa-miR-30c-2-3p	PM12646	10 nM	Applied Biosystems, Waltham, MA, USA
negative control miRNA #1	AM17010	10 nM	Applied Biosystems, Waltham, MA, USA
**Stealth RNAi siRNA**	**Assay ID**	**Concentration**	
siTOP2A	HSS110896 HSS186387	10 nM	Invitrogen, Waltham, MA, USA
**Antibody**	**Catalog Number**	**Dilution**	
Anti-*TOP2A*	12286	WB 1:1000 IHC 1:400	Cell Signaling Technology, Danvers, MA, USA
GAPDH	SAF6698	WB 1:2000	Wako, Osaka, Japan
Ki-67	M7240	IHC 1:100	Dako, Denmark, Denmark
HMGB2	ab124670	IHC 1:250	abcam, Cambridge, UK
SP1	9389	IHC 1:2000	Cell Signaling Technology, Danvers, MA, USA

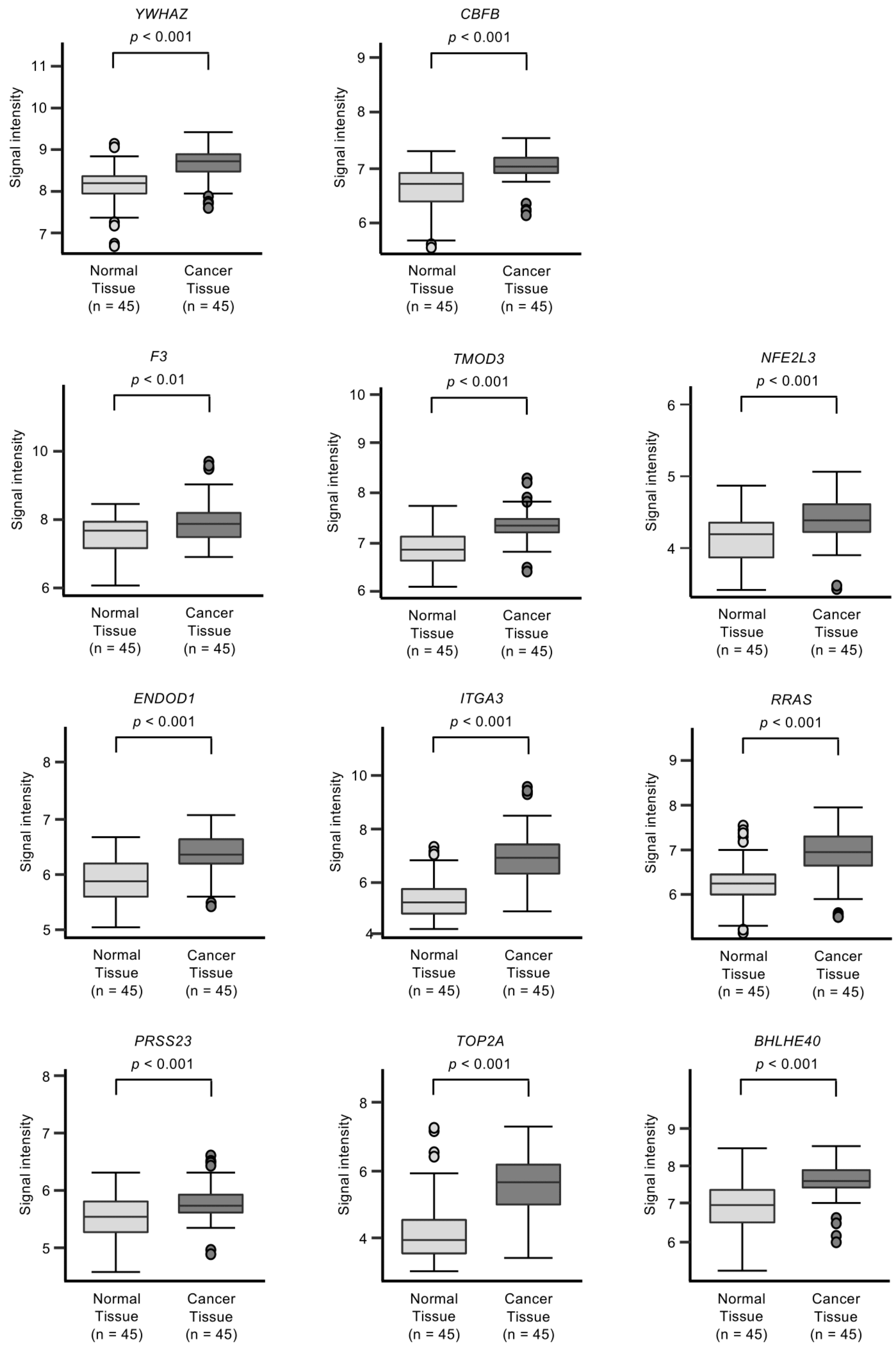
YWHAZ
p < 0.001
CBFB
p < 0.001
F3
p < 0.01
TMOD3
p < 0.001
NFE2L3
p < 0.001
ENDOD1
p < 0.001
ITGA3
p < 0.001
RRAS
p < 0.001
PRSS23
p < 0.001
TOP2A
p < 0.001
BHLHE40
p < 0.001
Signal intensity
Normal Tissue (n = 45)
Cancer Tissue (n = 45)

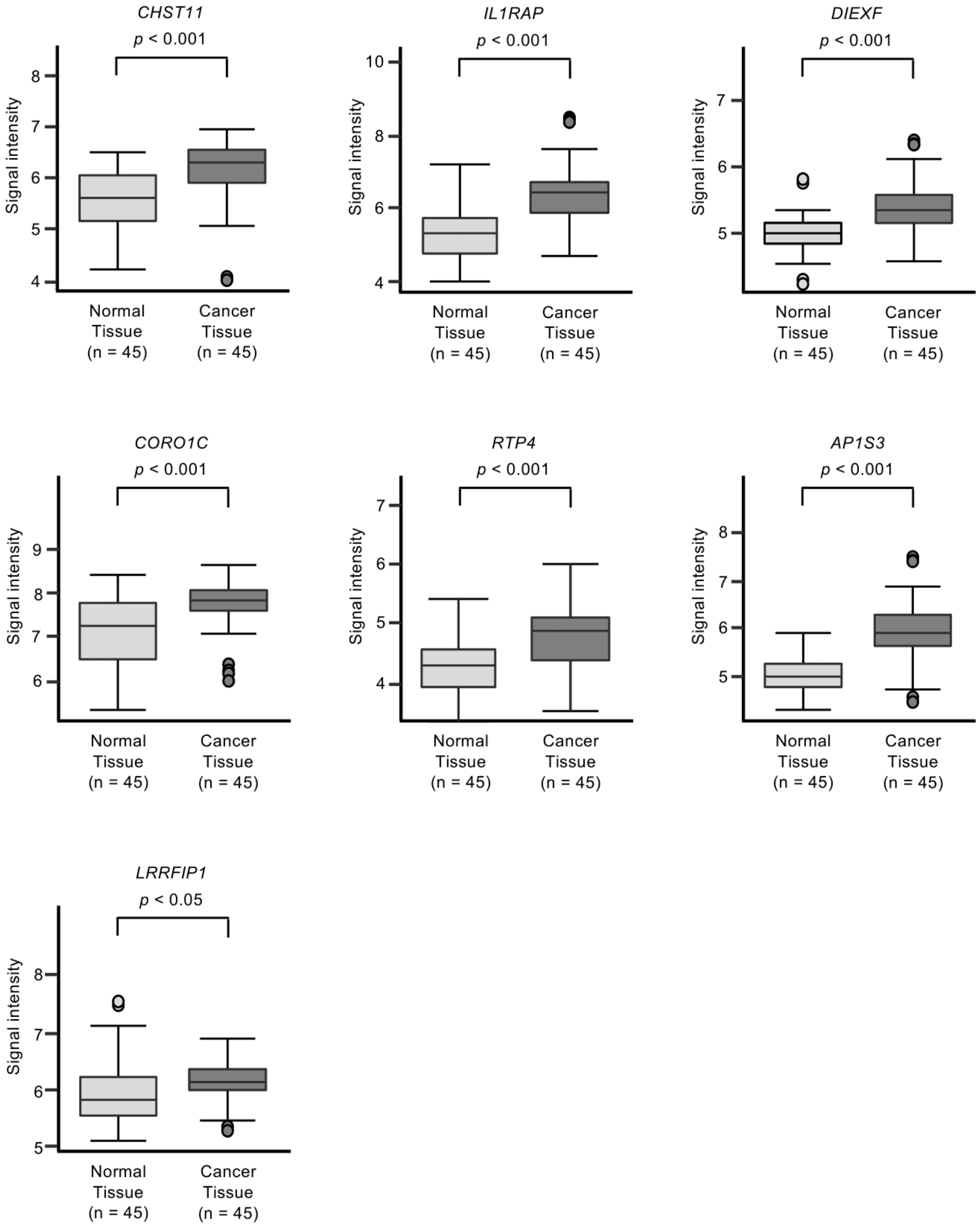

Figure S1. Expression levels of 18 target genes using GSE28735.

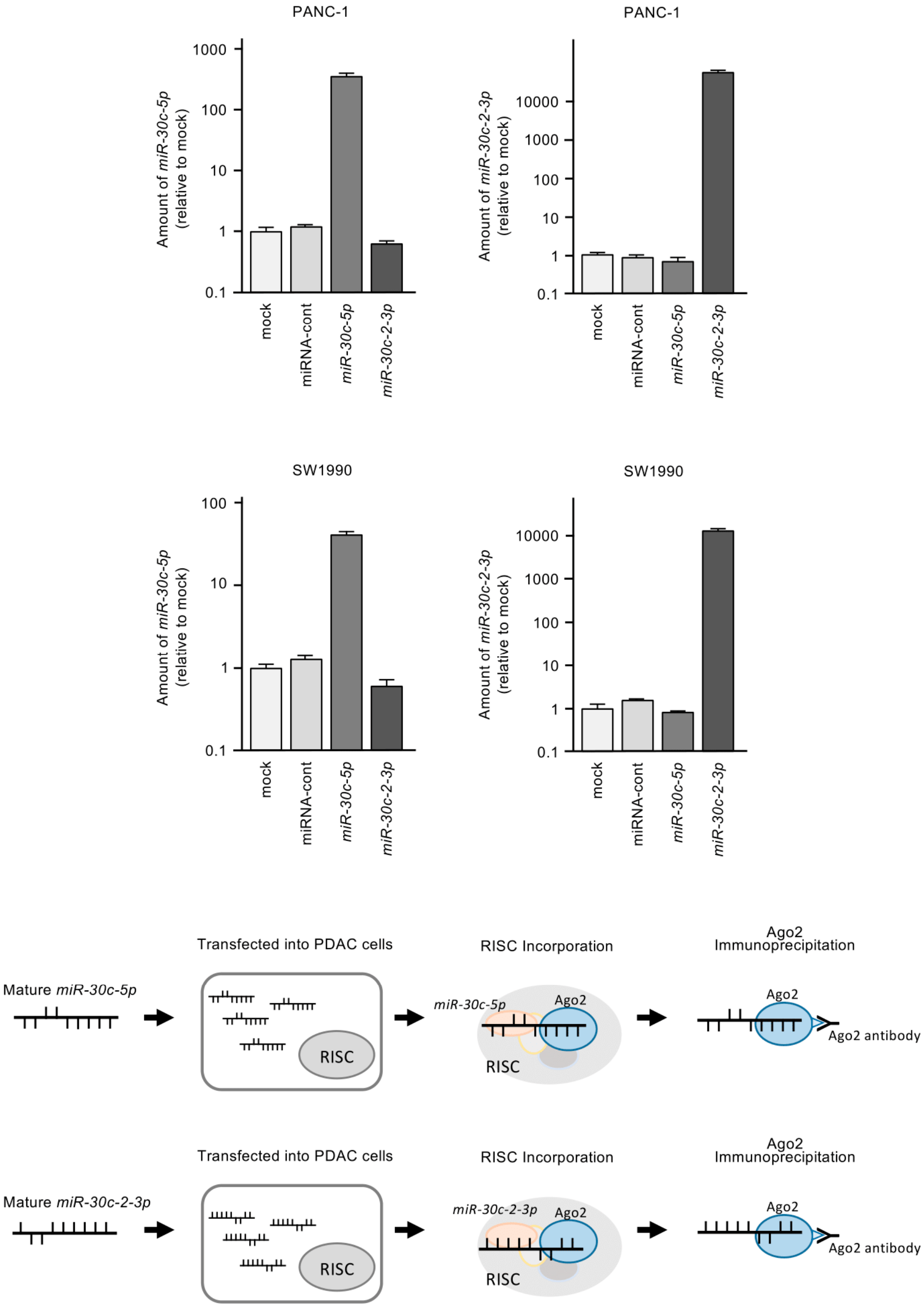

Figure S2. Incorporation of *miR-30c-5p* and *miR-30c-2-3p* into the RISC in PDAC cells.
